# Ionotropic glutamate receptor expression and function in the *Drosophila* testis stem cell niche

**DOI:** 10.17912/micropub.biology.002254

**Published:** 2026-08-05

**Authors:** Jennifer Viveiros, Neha Tripathi, Jasmine Grey, Brennan McDonald, Leif Benner, Brian Oliver, Erika Matunis

**Affiliations:** 1 Department of Cell Biology, Johns Hopkins University School of Medicine, Baltimore, MD, USA; 2 Department of Biology, Stanford University, Stanford, CA, USA; 3 Department of Genetics, Harvard Medical School, Boston, MA, USA; 4 The O’Neill School of Public and Environmental Affairs, Indiana University, Bloomington, IN, USA

## Abstract

Adult stem cells are required to maintain tissue homeostasis and understanding their regulation is essential to leveraging their utility. Here we report that seven transgenic ionotropic glutamate receptor (iGluR) subunit reporter lines are variably expressed in hub cells of the *Drosophila* testis stem cell niche, suggesting a novel form of inter-organ communication for this tissue. While HCR-FISH confirms that one of the iGluR subunits, *GluRIIA* , is transcribed in hub cells, we do not detect GluRIIA protein expression. Furthermore, hub cells are not sensitive to exogenous glutamate and hub-specific knockdown of the essential iGluR subunits does not phenotypically affect cells of the testis apex. Altogether this suggests that iGluRs are not essential for stem cell niche homeostasis.

**Figure 1. Glutamate receptor enhancers are active, but receptors are not expressed in hub cells:** (A – B) Illustration of the *Drosophila* (A) testis and (B) the testis apex. The testis is a coiled blind ended tubule in which the germline cells (purple) exit the apex as they differentiate, ultimately becoming sperm (grey). The testis wall is composed of an outer pigment cell layer (pink) and inner muscle cell layer (orange) with basal laminae underlying each layer. At the testis apex, the niche cells (hub, green) maintain germline stem cells (GSCs, dark purple) and cyst stem cells (CySCs, dark blue). As the stem cells divide, their progeny move away from the hub and differentiate. GSC daughters, or gonialblasts, undergo four rounds of transit amplification (spermatogonia, light purple). CySCs divide and give rise to quiescent daughter cyst cells (light blue) that wrap and support adjacent germ cells. (C – P) Representative (C – I) single confocal sections through testis apices or (J – P) maximum intensity projections of testes from (C,J) *GluRIA* , (D,K) *GluRIB* , (E,L) *GluRIIA* , (F,M) *GluRIIB* , (G,N) *GluRIIC* , (H,O) *GluRIID* , or (I,P) *GluRIIE* expression reporter flies. Testes were stained for enhancer reporter expression (V5, green; insets), nuclei (DAPI, blue), and (C – I) hub cell membranes (N-cadherin (N-Cad), magenta). (Q) Representative single confocal section through Oregon-R testis apex labeled using HCR-FISH probes against *upd1* (orange) and *GluRIIA* (green; inset) mRNA transcripts and counterstained with DAPI (nuclei, blue). (R – S) Representative single confocal sections through Oregon-R (R) testis wall or (S) testis apex stained for GluRIIA (green; inset), basement membranes (Concanavalin A, magenta), and nuclei (DAPI, blue). (T) Representative single confocal section through *GluRIIA-GAL4 > UAS-RedStinger* testis apex stained for GAL4 expression (DsRed, magenta; inset), hub cell membranes (N-Cad, green), and nuclei (DAPI, blue). (U) Quantification of relative GCaMP signal within the hub of explanted control testes or testes treated with 1 mM L-glutamate over time (vertical line indicates frame timing of L-glutamate addition). For each testis, GCaMP signal was normalized to the mean fluorescence intensity of the first frame. 10 testes were imaged per condition. Scale bars, (C – I, Q – T) 10 µm or (J – P) 100 µm. Hubs outlined (dashed white lines). Insets display fluorescent signals in greyscale.

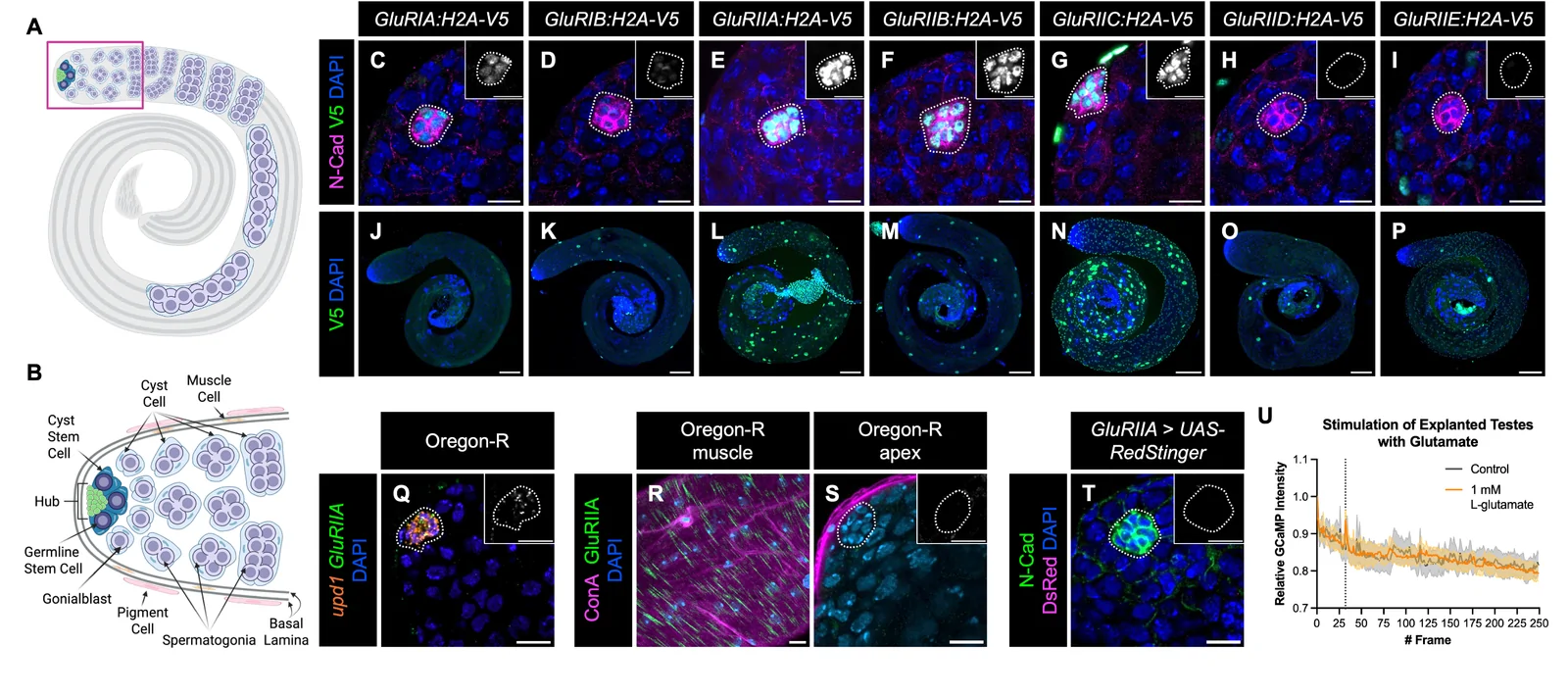



## Description

Adult stem cells are critical to the maintenance and regeneration of tissues. By using model systems like that of the *Drosophila* testis stem cell niche, we can better understand the cellular and molecular mechanisms that regulate stem cell behaviors. Adult stem cells reside in dynamic microenvironments, or niches, that are composed of cellular and molecular constituents including extracellular matrix and support cells (reviewed in (Morrison and Spradling, 2008)). The *Drosophila* testis is a blind-ended tubule that houses a single stem cell niche located at its apex ( **Fig. 1A** ). This stem cell niche maintains two stem cell populations, the germline stem cells (GSCs) and the somatic cyst stem cells (CySCs), both of which reside in direct contact with somatic niche, or hub, cells ( **Fig. 1B** ). The hub acts as both a signaling center and an adhesion platform to maintain both stem cell populations (reviewed in (de Cuevas and Matunis, 2011; Siddall and Hime, 2017)). Therefore, gaining insight into the hub is fundamental to our understanding of the biology of this stem cell niche.

Ionotropic glutamate receptors (iGluRs) are heterotetrameric channels that facilitate neuromuscular junction activity in response to the primary neurotransmitter glutamate (DiAntonio et al., 1999; Han et al., 2015; Han et al., 2024; Jan and Jan, 1976). Following an action potential, motor neurons release glutamate into the post-synaptic space, where it binds to the iGluRs on muscle cells, allowing the influx of calcium to trigger muscle contraction (Schuster et al., 1991; Takeuchi and Takeuchi, 1963; Takeuchi and Takeuchi, 1964). In mammals, glutamate acts as the primary neurotransmitter in the brain and its concentration is tightly regulated via a gradient maintained by the blood brain barrier; the concentration of glutamate is low in the brain and high in the blood (Vandenberg and Ryan, 2013). Similarly, *Drosophila* hemolymph contains high concentrations of glutamate, which may act as its source for the testis apex (Chen et al., 2009; Echalier, 1997; Fairchild et al., 2016). Furthermore, glutamatergic neurons innervate the internal male reproductive system, including the base of the testis, to coordinate copulation (Chaverra et al., 2025; Pavlou et al., 2016). Surprisingly, previous single-nuclei RNA sequencing of the testis detected mRNA transcripts encoding kainate-type and ⍺-amino-3-hydroxy-5-methyl-4-isoxazolepropionic (AMPA)-like iGluRs subunits (Li et al., 2016) in the hub cells, a population of cells not thought to express such proteins (Li et al., 2022; Mahadevaraju et al., 2021; Raz et al., 2023). Therefore, the presence of iGluR transcripts within the niche suggests an avenue for inter-organ communication.

To begin our characterization of iGluR expression in the testis, we generated enhancer reporter transgenic fly strains for kainate-type ( *GluRIIA* , *GluRIIB* , *GluRIIC* , *GluRIID* , and *GluRIIE* ) and AMPA-like ( *GluRIA* and *GluRIB* ) iGluR subunits (see methods for details). Kainate-type iGluRs are tetramers consisting of three obligate subunits (GluRIIC, GluRIID, and GluRIIE) combined with one of two variable subunits (GluRIIA and GluRIIB) (Featherstone et al., 2005; Marrus et al., 2004; Qin et al., 2005). We examined testes of each transgenic fly and found that hub cell expression of iGluR subunits is highly variable, with some reporters showing high expression and others displaying little to none. Within the hub, reporters of *GluRIIA* ( **Fig. 1E** ), *GluRIIB* ( **Fig. 1F** ), and *GluRIIC* ( **Fig. 1G** ) were among the most highly expressed, while *GluRIA* ( **Fig. 1C** ) and *GluRIB* reporters ( **Fig. 1D** ) were expressed at moderate levels. Reporters of *GluRIIE* ( **Fig. 1I** ) and *GluRIID* ( **Fig. 1H** ) showed little to no expression in the hub cells. Reporter activity was not restricted to hub cells but also marked the muscle cells and pigment cells in a strain-specific manner. For instance, *GluRIIA* ( **Fig. 1L** ) and *GluRIIC* ( **Fig. 1N** ) were highly expressed in both muscle and pigment cells, *GluRIIE* ( **Fig. 1P** ) was predominantly expressed in muscle cells, and *GluRIB* ( **Fig. 1K** ), *GluRIIB* ( **Fig. 1M** ), and *GluRIID* ( **Fig. 1O** ) were expressed in pigment cells, while *GluRIA* ( **Fig. 1J** ) was not detected in either cell population. The variation between *GluRIIC* , *GluRIID* , and *GluRIIE* reporter expression was surprising, as these subunits are all necessary to form the final heterotetrameric protein complexes. Furthermore, estimates of transcript abundance in larval body wall tissue suggest that *GluRIID* and *GluRIIE* transcripts are more abundant than those of *GluRIIC* (Qin et al., 2005), but expression patterns of our reporters revealed the opposite relationship in the hub cells. However, it should be noted that the reporter fly strains may not fully reflect the endogenous expression patterns of each gene due to their design (see methods for details). Altogether, our analysis of the iGluR subunit expression reporter fly lines suggests that both AMPA-like iGluR subunits are detectable in hub cells, whereas the full complement of kainite-type iGluR subunits is not.

Expression of enhancer reporter elements does not necessarily mean that the respective proteins are expressed. To investigate whether iGluR gene products are produced in hub cells, we assessed both transcript and protein expression of GluRIIA, the subunit with the strongest enhancer reporter expression. Using HCR-FISH, we observed that *GluRIIA* mRNA is detectable in hub cells, which were co-labeled with probes against the stemness-conferring signaling ligand *upd1* ( **Fig. 1Q** ) (Kiger et al., 2001; Tulina and Matunis, 2001). Further, using antisera against GluRIIA, we detected GluRIIA protein in the muscles of the testis wall, which is consistent with their contractile role ( **Fig. 1R** ). However, we did not observe GluRIIA protein expression in hub cells ( **Fig. 1S** ). To verify the absence of GlurIIA protein in hub cells, we examined the expression pattern of a *GluRIIA-T2A-GAL4* driver line, which produces GAL4 as a separate protein under endogenous GluRIIA control (Deng et al., 2019). We surveyed testes from *GluRIIA-T2A-GAL4 > UAS-RedStinger* flies for RedStinger expression in hub cells and apart from one hub cell (n = 1/40 testes), we did not detect GAL4 activity in the hub ( **Fig. 1T** ). Altogether, this suggests that iGluR genes are transcribed in hub cells, but that the corresponding proteins are not produced, indicating potential post-transcriptional or post-translational regulation of iGluR subunit genes. Analogous de-coupling of iGluR subunit gene transcription and translation has also been observed during *Drosophila* embryogenesis (Ganesan et al., 2011).

Given the inconsistency between transcript expression and lack of detectable GluRIIA protein in hub cells, we investigated whether iGluRs have a functional role in the testis stem cell niche. To measure hub cell reactivity to glutamate, we live imaged testes expressing a fluorescent calcium reporter, GCaMP6s (Parkhurst, 2020), specifically in hub cells using the hub specific driver, *E132-GAL4* (Kawase et al., 2004). At the onset of imaging, GFP fluorescence, the calcium reporter signal, was detected in all the hub cells. However, we did not observe any change in GFP fluorescence signal following addition of 1 mM L-glutamate to the imaging media (Huang et al., 2019), and relative GCaMP intensity plots were similar to control testes ( **Fig. 1U** ). This suggests that hub cells are not responsive to glutamate. We also asked whether iGluR expression in hub cells is necessary to support the stem cell niche, which could suggest that iGluRs have a non-excitatory role in the hub (reviewed in (Featherstone, 2010)). We hypothesized that if hub cells need iGluRs to coordinate stem cell niche homeostasis, we would observe a loss of cells in the apices of testes with reduced iGluR expression in hub cells. Using *E132-GAL4* , we expressed RNAi transgenes targeting the essential subunits, GluRIIC, GluRIID, or GluRIIE, and assayed for differences in cellular composition within the testis apex relative to testes expressing the control RNAi transgene ( *UAS-mCherry RNAi* ). In testes with hub cell-specific knockdown (KD) of GluRIIC, GluRIID, or GluRIIE we observed hub cells (Fasciclin 3-positive), germ cells (Vasa-positive) and early cyst cells (Zfh1-positive) in comparable abundance to control testes (GluRIIC KD: 61/61 testes; GluRIID KD: 62/62 testes; GluRIIE KD: 93/95 testes, Fisher’s exact test p = 0.4975; mCherry KD: 91/91 testes). Of the two outlier testes from the GluRIIE KD flies, one had fewer germ cells than control testes (n = 1/95) and one had a germline tumor (n = 1/95). Overall, these results indicate that iGluRs likely do not function in hub cells to maintain homeostasis of this stem cell niche. A caveat of this result is that the RNAi-mediated knockdown may not have sufficiently reduced protein abundance. However, these same RNAi transgenes have been shown to effectively produce loss-of-function phenotypes in other tissues (Sulkowski et al., 2014). We also cannot rule out the possibility that reduced iGluR function affects the testis stem cell niche in more subtle ways (i.e. reduced stem cell division rate). Finally, iGluRs may be necessary under varying environmental or physiological conditions not assayed here, including starvation, aging, and excessive mating.

Through this work we have demonstrated that reporters of iGluR gene expression are variably detected in hub cells as well as pigment cells and muscle cells of the testis. While we confirmed that transcripts of one iGluR subunit, GluRIIA, are detectable in hub cells, we did not detect respective protein expression in hub cells, although GluRIIA expression in the muscle cells of the testis wall confirmed that the antisera is functional. This absence of protein expression was reflected in a lack of response to exogenous glutamate and the absence of deleterious effects following hub-specific knockdown of essential kainate-type iGluR subunits. This work demonstrates that despite transcriptional activity, iGluRs are not expressed in hub cells and do not function in hub cells to maintain testis homeostasis. Future work will need to be performed to investigate the role of iGluRs in the muscle cells and pigment cells of the testis.

## Methods

**Fly husbandry and stocks**

Flies were maintained on a standard yeast/cornmeal/molasses medium supplemented with dry yeast, as previously described (Greenspan et al., 2022). Stocks were kept at 25°C and crosses were set and matured at 25°C. *GluRIIA-T2A-GAL4 > UAS-RedStinger* males were collected at 0 – 4 days old and transferred to 29°C for 4 days to enhance GAL4 driver expression. Male flies less than 8 days old were used for all experiments. See table of Reagents for list of fly stocks. We used FlyBase (release FB2025_05) to find information on phenotypes/function/stocks/gene expression (Öztürk-Çolak et al., 2024).

**Generation of transgenic fly lines**

Enhancer reporter lines of *GluRIA* , *GluRIB* , *GluRIIA* , *GluRIIB* , *GluRIIC* , *GluRIID* , and *GluRIIE* were generated as previously described (Mahadevaraju et al., 2024). For each gene, the 1.25 kb region upstream and 250 bp region downstream of the transcriptional start site was cloned and inserted upstream of the basal P-element promoter driving expression of the histone H2A coding sequence and three V5 tag sequences. Injections of transgenes were performed by BestGene (Chino Hills, CA).

**Testis dissection and immunofluorescence**

Testis dissection and immunofluorescence were performed as previously described (Matunis et al., 1997; Viveiros and Matunis, 2026). Unless noted, all steps were executed at room temperature (RT), and a nutating platform was used for all incubation steps. CO _2_ -anesthetized male flies were dissected in 1X Ringer’s solution. Testes with attached cuticle were transferred to fixative (4% paraformaldehyde in 1X PBS with 0.1% Triton X-100 (1X PBX)) and incubated for 20 min. Fixed testes were rinsed twice and washed for 30 min in 1X PBX and then incubated in block solution (3% BSA and 0.02% NaN _3_ in 1X PBX) supplemented with 2% normal goat serum (Millipore/Sigma, G9023) for one hour at RT or overnight at 4°C. Testes were then transferred to primary antibodies diluted in block and incubated overnight at 4°C, followed by two rinses, and an hour wash in 1X PBX, and then incubated in secondary antibodies and 4,6-diamidino-2-phenylindole (DAPI; 1 μg/mL, Millipore/Sigma, 10236276001) diluted in block for 1.5 hrs at room temperature or overnight at 4°C. Testes were rinsed briefly then washed for an hour in 1X PBX. Finally, to remove detergent, testes were rinsed and washed for 10 min in 1X PBS before transferring to Vectashield (Vector Laboratories, H-1000). Samples were stored at -20°C prior to imaging. Primary antibodies used were against Fasciclin 3 (1:50, DSHB, 7610), N-cadherin (N-Cad; 1:20, DSHB, DN-Ex #8), GluRIIA (1:50, DSHB, 8B4D2), Vasa (1:20, DSHB), Zfh1 (1:500, our lab), V5 tag (1:2,000, Thermo Fisher Scientific, R960-25), and DsRed (1:5,000, Takara Bio, 632496). Secondary antibodies used were Goat anti-Mouse IgG (H+L) Highly Cross-Adsorbed Secondary Antibody, Alexa Fluor™ 488 (1:200, Thermo Fisher Scientific, A-11029), Goat anti-Mouse IgG (H+L) Cross-Adsorbed Secondary Antibody, Alexa Fluor™ 488 (1:200, Thermo Fisher Scientific, A-11001), Goat anti-Rat IgG (H+L) Cross-Adsorbed Secondary Antibody, Alexa Fluor™ 488 (1:200, Thermo Fisher Scientific, A-11006), Goat anti-Rabbit IgG (H+L) Cross-Adsorbed Secondary Antibody, Alexa Fluor™ 568 (1:100, Thermo Fisher Scientific, A-11011), Goat anti-Rat IgG (H+L) Cross-Adsorbed Secondary Antibody, Alexa Fluor™ 568 (1:100, Thermo Fisher Scientific, A-11077), Goat anti-Guinea Pig IgG (H+L) Highly Cross-Adsorbed Secondary Antibody, Alexa Fluor™ 568 (1:100, Thermo Fisher Scientific, A-11075), and Goat anti-Mouse IgG (H+L) Highly Cross-Adsorbed Secondary Antibody, Alexa Fluor™ 647 (1:66, Thermo Fisher Scientific, A-21236). Basement membranes were stained with Concanavalin A, Alexa Fluor™ 594 conjugate (1:50, Thermo Fisher Scientific, C-11253).

**Hybridization Chain Reaction-Fluorescence In Situ Hybridization (HCR-FISH)**

Testes were processed for HCR-FISH using Molecular Instruments HCR (v3.0) buffers, hairpins, and probes and an adapted version of a previously described protocol (Grmai et al., 2024; Slaidina et al., 2020). All steps were performed on a nutator at room temperature unless otherwise noted. Testes from Oregon-R males were dissected in 1X PBS on ice then moved to an Eppendorf tube containing 1X PBS on ice after every five pairs of testes for up to 30 min. Testes were fixed in 4% paraformaldehyde in 1X Dulbecco’s Phosphate Buffered Saline (DPBS) with 0.1% Tween-20 (DPBS-Tw) for 20 min and then washed 2 x 5 min in DPBS-Tw. Tissues were then dehydrated in sequential washes with 25%, 50%, 75%, and 100% MeOH in DPBS-Tw for 5 min each on ice prior to storage at -20°C overnight. Testes were rehydrated with sequential washes with 75%, 50%, 25%, and 0% MeOH in DPBS-Tw for 5 min each on ice, followed by a 2 hr permeabilization with 1% Triton-X in 1X DPBS, and a 20 min post-fix in 4% paraformaldehyde in DPBS-Tw. After fixation, testes were washed 2 x 5 min DPBS-Tw, 1 x 5 min 50%/50% DPBS-Tw/5X SSC with 0.1% Tween-20 diluted in 1X DPBS (SSC-Tw), and 2 x 5 min SSC-Tw, on ice. Tissues were pre-hybridized in probe hybridization buffer (Molecular Instruments) for 30 min at 37°C and then incubated in diluted probes (0.8 pmol in 1 mL in probe hybridization buffer) for 12-16 hrs at 37°C. Following hybridization, testes were washed 4 x 15 min at 37°C with probe wash buffer warmed to 37°C (Molecular Instruments), and then with SSC-Tw for 2 x 5 min at room temperature. To equilibrate, testes were incubated in amplification buffer for 10 min at room temperature (not nutating). Hairpin solutions were prepared by heating 6 pmol of each hairpin (B3 488 and B1 546 HCR™ Amplifier (v3.0)) for 90 seconds at 95°C, snap cooling at room temperature in the dark for 30 min, and then adding to 100 μL of room temperature amplification buffer (Molecular Instruments). Testes were incubated in hairpin solutions overnight at room temperature followed by washes in SSC-Tw: 2 x 5 min on benchtop, 2 x 30 min, and 1 x 5 min. DAPI (1:1000) was added to the final 30 min SSC-Tw wash during the last 15 min. Detergent was removed by washing in 1X PBS for 5 min. Testes were stored briefly in Vectashield prior to mounting (same day).

**Microscopy and image analysis**

Testes were mounted on standard slides under #1.5 thickness glass coverslips. Images were obtained using a Zeiss LSM 800 microscope equipped with a 63x oil immersion objective, 405 nm, 488 nm, 561 nm, and 640 nm diode lasers with digital zoom, and GaAsP and Airyscan detectors. Images were acquired using Zen software with Z-stacks acquired using a 0.5 μm step, followed by processing using Zen or FIJI (Schindelin et al., 2012). Brightness for individual channels from single confocal slices was enhanced using Zen or FIJI, and then the channels were overlaid to form a merged image. Single slices and maximum intensity projections are shown in this paper as indicated.

**Live imaging of testes**

*E132-GAL4 > UAS-GCaMP6s* testes were dissected, cultured, and live imaged as previously described (Greenspan and Matunis, 2023) using a Zeiss LSM 900 confocal microscope. Experiment design was adapted from previous calcium imaging performed in explanted testes (Martin-Diaz and Herrera, 2024). Two testes of each dish (10 dishes total) were designated as either control or experimental. Testes were imaged for 10 minutes with frames approximately every 2.5 seconds (variability due to definite focus). For each dish, the control testis was imaged first, followed by imaging of the experimental testis. For the experimental testis, 1 mM L-glutamate (diluted in live imaging solution; (Huang et al., 2019)) was added to the dish after 32 frames were collected.

**Quantifications and statistical analysis**

FIJI was used to determine the *E132-GAL4 > UAS-GCaMP6s* fluorescence intensity measurements. A region of interest was manually drawn around the hub, and the mean grey value of that region was acquired. Measurements of each frame were normalized to that of the first frame of the same testis.

All statistical analysis and generation of graphical representation were performed using GraphPad Prism 11.

## Reagents

**Table:** 

**Strain**	**Genotype**	**Source or Reference**
Oregon-R	Oregon-R	Gift of D. Andrew
*GluRIA:H2A-V5*	*y[1] w[1118]; P{y[+] DsRed[+]=GluRIA[Enhancer]-PeleP-H2A-3xV5}attP40/CyO*	This paper
*GluRIB:H2A-V5*	*y[1] w[1118]; P{y[+] DsRed[+]=GluRIB[Enhancer]-PeleP-H2A-3xV5}attP40/CyO*	This paper
*GluRIIA:H2A-V5*	*y[1] w[1118]; P{y[+] DsRed[+]=GluRIIA[Enhancer]-PeleP-H2A-3xV5}attP40/CyO*	This paper
*GluRIIB:H2A-V5*	*y[1] w[1118]; P{y[+] DsRed[+]=GluRIIB[Enhancer]-PeleP-H2A-3xV5}attP40/CyO*	This paper
*GluRIIC:H2A-V5*	*y[1] w[1118]; P{y[+] DsRed[+]=GluRIIC[Enhancer]-PeleP-H2A-3xV5}attP40/CyO*	This paper
*GluRIID:H2A-V5*	*y[1] w[1118]; P{y[+] DsRed[+]=GluRIID[Enhancer]-PeleP-H2A-3xV5}attP40/CyO*	This paper
*GluRIIE:H2A-V5*	*y[1] w[1118]; P{y[+] DsRed[+]=GluRIIE[Enhancer]-PeleP-H2A-3xV5}attP40/CyO*	This paper
*UAS-RedStinger*	*w[1118]; P{w[+mC]=UAS-RedStinger}6*	BDSC_8547
*GluR-T2A-GAL4*	w[*]; TI{2A-GAL4}GluRIIA[2A-GAL4]	BDSC_84637
*E132-GAL4*	P{w[+mW.hs]=GawB}E132, w[*]	BDSC_26796
*GCaMP6s*	w[*]; P{w[+mC]=UASp-GCaMP6s}30/TM3, Sb[1]	BDSC_91366
*GluRIIC RNAi*	*y[1] v[1]; P{y[+t7.7] v[+t1.8]=TRiP.JF01854}attP2*	BDSC_25836
*GluRIID RNAi*	*y[1] v[1]; P{y[+t7.7] v[+t1.8]=TRiP.JF02035}attP2*	BDSC_26010
*GluRIIE RNAi*	*y[1] v[1]; P{y[+t7.7] v[+t1.8]=TRiP.JF01962}attP2*	BDSC_25942
*mCherry RNAi*	y[1] sc[*] v[1] sev[21]; P{y[+t7.7] v[+t1.8]=VALIUM20-mCherry.RNAi}attP2	BDSC_35785

**Table:** 

**HCR probe**	**Description**	**Source**
*upd1*	Fruit fly *upd1* HCR v3.0 probe set (B1) set size: 20	Molecular Instruments
*GlurIIA*	Fruit fly *GlurRIIA* HCR v3.0 probe set (B3) set size: 20	Molecular Instruments
